# The Exogenous Application of Micro-Nutrient Elements and Amino Acids Improved the Yield, Nutritional Status and Quality of Mango in Arid Regions

**DOI:** 10.3390/plants10102057

**Published:** 2021-09-29

**Authors:** Ahmed M. S. Kheir, Zheli Ding, Mohamed S. Gawish, Hanan M. Abou El Ghit, Taghred A. Hashim, Esmat F. Ali, Mamdouh A. Eissa, Zhaoxi Zhou, Mohammad S. Al-Harbi, Sherif Fathy El-Gioushy

**Affiliations:** 1 Haikou Experimental Station, Chinese Academy of Tropical Agricultural Sciences (CATAS), Haikou 570000, China; ahmedkheir@arc.sci.eg; 2 Agricultural Research Center, Soils, Water and Environment Research Institute, Giza 12112, Egypt; 3 Pomology Department, Faculty of Agriculture, Damietta University, Damietta 34511, Egypt; msagawishaa@gmail.com; 4 Botany and Microbiology Department, Faculty of Science, Helwan University, Cairo 11111, Egypt; hanan8760@yahoo.com; 5 Soil and Water Department, Faculty of Agriculture (Moshtohor), Benha University, Moshtohor, Toukh 13736, Egypt; taghreed.hashem@fagr.bu.edu.eg; 6 Biology Department, Faculty of Science, Taif University, Taif 26571, Saudi Arabia; a.esmat@tu.edu.sa (E.F.A.); mharbi@tu.edu.sa (M.S.A.-H.); 7 Department of Soils and Water, Faculty of Agriculture, Assiut University, Assiut 71526, Egypt; mamdouh.eisa@aun.edu.eg; 8 Horticulture Department, Faculty of Agriculture (Moshtohor), Benha University, Moshtohor, Toukh 13736, Egypt

**Keywords:** mango, Fagri Kalan, micronutrients, foliar application, amino acids, nutritional status, fruiting aspects

## Abstract

The mango is one of the most valuable and appealing tropical fruits due to its color, aroma, tasteful remarkable flavor, and nutritive value; however, improving the yield and quality of mango is an urgent goal in order to combat global population growth. The application of amino acids and a micronutrient mixture might improve the yield and quality features but further research is still required in arid regions. To study the combined effect of a micronutrient mixture (MM) and amino acids (AA) at different rates, twenty-seven Fagri Kalan mango trees (15 years old) were carefully selected. The foliar application effect of MM and AA on vegetative growth, total chlorophyll, leaf chemical constituents, productivity, and the fruit quality of mango trees (cv. *Fagri Kalan*) was investigated. The findings revealed that the investigated growth measurements and leaf chemical contents, as well as the fruiting aspects and the fruit quality improved significantly due to the application of MM and AA. A higher application rate of the micronutrient mixture (2 g L^−1^) in combination with the highest amino acid concentration (2 mg L^−1^) was the most effective combination that increased the yield, total soluble solids (TSS), total sugars (TS), and total carbohydrates by 28.0%, 3.0%, 5.8% and 15.0%, respectively, relative to untreated plants. The relationship between such characteristics revealed a strong positive correlation (0.80–0.95), confirming the importance of these materials in increasing the yield and quality of mangoes. Thus, using doses of MM and AA as a foliar spray four times during each growing season is recommended under similar environmental conditions and horticulture practices used in the current experiment.

## 1. Introduction

The mango (*Mangifera indica* L.) is one of the most common and one of the oldest cultivated fruits in tropical and subtropical regions spanning over 100 countries [1,2]. It is the second most grown tropical fruit and the sixth most important fruit crop worldwide [3], showing a high resistance to climate change [4]. It is also considered one of the most valuable and attractive tropical fruits due to its color, aroma, tasteful and remarkable flavor, and nutritive value. In addition, it is an excellent source of carotenoids, vitamins C, A, E, B, riboflavin, niacin, thiamin, phenolics, carbohydrates and minerals such as Fe, P, Ca, and K [5]. Mango fruits could thus be regarded as a suitable food source for current and future population growth. Mango orchards, on the other hand, confront a slew of issues, including micronutrient deficiency, physiological stress, and fruit yield and quality issues, all of which reduce output and exports [6]. Fertilization with micro and macronutrients has a significant impact on yield productivity and fruit quality [7]. To our knowledge, little emphasis has been placed on the optimum nutrient application that alleviates plant micronutrient shortages through foliar or soil application while also improving fruit quality attributes and yield.

The majority of African countries, including Egypt, are suffering from intensified intercropping, low fertility, and inefficient fertilizer application [8], resulting in a severe lack of micronutrients, particularly iron and zinc [9]. Micronutrients are known to be necessary for metabolic activities, enzymatic reactions, and redox reactions in plant cells, as well as for the synthesis of amino acids [10]. This issue is very common in calcareous soils due to the limited replenishment of fertilizers, and intensive intercropping which causes a long-term deficiency of micronutrients.

Amino acids (AA) are bio stimulants because they encourage plant growth and they enhance the quality and the nutritional status of plants [11,12,13]. The application of AA not only improves abiotic stress mitigation [14], but it also serves as a hormone precursor [11,13,15,16], enables distinct physiological progression signaling factors, regulates N uptake [17], and promotes root growth and development [18,19], and antioxidant metabolism [20,21]. Furthermore, the application of amino acids could increase the K+ content in plants even under abiotic stress or in normal conditions [21]. The foliar application of AA mixtures has recently demonstrated beneficial effects on plants, including but not limited to the increased production of Solanum lycopersicum [22], chlorophyll [23], dry matter, polysaccharides, and starch in *Vicia faba* L. [24]. However, no additional research has been conducted to investigate the effect of AA foliar application on the yield and quality of mangoes in arid and semi-arid conditions. Furthermore, the incorporation of micronutrients and amino acids has received less attention thus far, confirming the significance of the current study.

Therefore, the main objective of this study was to explore the exogenous effect of micronutrients and amino acid mixtures on the yield, quality and elemental content in terms of nutrition for Fagri Kalan mango trees. The best concentration of amino acids and micronutrients that could ensure a higher yield and quality in arid regions was also investigated.

## 2. Results

### 2.1. Vegetative Growth and Nutritional Status

The obtained results show a significant increase in vegetative traits such as shoot length, shoot diameter, leaf number, and leaf area (Tables S1 and S2) associated with an increase in the rates of the foliar application of amino acids and the micronutrient mixture. The application of foliar amino acids resulted in a significant increase in such vegetative parameters as the application rate increased, reaching a maximum significant increase by applying A2. The shoot length increased by 5.9% and 9.5% in the first season and 5.5% and 9.8% in the second season under A1 and A2, respectively, whilst the increase in shoot diameter was 7.2% and 15% in the first season and 7.2% and 14.7% in the second season due to A1 and A2, respectively. The results shown in Table S2 show that the number of leaves/shoots increased by 7.6% and 12.1% in the first season and 9.1% and 13.9% in the second season with the application of A1 and A2, respectively. Increasing the amino acid rate significantly affected the number of new shoots in both seasons. Similarly, spraying a micronutrient mixture resulted in a significant increase in shoot length, shoot diameter, the number of leaves, the leaf area, and the total chlorophyll with the largest increase related to the highest rate of application. In terms of the interaction effect between the application of the amino acids and the micronutrient mixture, the obtained results show that increasing the application rates of amino acids in the absence and presence of a micronutrient mixture increased the parameters of vegetative growth significantly. Likewise, increasing the rates of the micronutrient mixture resulted in a progressive significant increase in vegetative growth related to the levels of amino acids. The highest values in relation to the shoot length were 57.40 cm and 58.41 cm, which were achieved with the application of A2M2, while the lowest ones were 44.03 cm and 44.86 cm noted in the control (A0M0) with increases of 30.4% and 30.2% in the first and second season, respectively. The same trend was seen with other vegetative parameters such as shoot diameter, the number of new shoots, the number of leaves per shoot, and the leaf area, confirming the importance of using higher M and AA rates.

In terms of the nutritional status of the mangoes, the foliar application of amino acids resulted in a significant increase in the plant content of chlorophyll and the total carbohydrates (Table 1), nitrogen, phosphorus, potassium (Table S3), magnesium (Table S4), iron, zinc, and manganese (Table S5) due to the increased application rate of the amino acids, but it had no effect on calcium content (Table S4). The total chlorophyll increased significantly by 4.5% and 9.2% in the first season, while the increase in the second season reached up to 3.2% and 7.6% with the application of A1 and A2, respectively (Table 1). The most effective rate was 2 mL L^−1^ (A2) in both seasons, which achieved the highest content of total carbohydrates by 5.0 and 4.7% in the first and second seasons, respectively.

In terms of the effect of spraying the micronutrient mixture, increasing the application rate of the micronutrient mixture resulted in a significant increase in the plant content of chlorophyll, nitrogen, phosphorus, potassium, magnesium, iron, zinc, and manganese, but it had no effect on the plant content of calcium. The total chlorophyll increased significantly by 16.4% and 24.3% in the first season and increased by 16.6% and 26.9% in the second season due to M1 and M2, respectively. Likewise, the total carbohydrates increased by 14% with the application of M2. The results also show that the nitrogen content increased by 10.3% and 15% in the first season and 11.6% and 15.7% in the second season in response to the application of M1 and M2, respectively. Similar improvements were achieved with other minerals except Ca (Tables S3–S5). The overall view is that the nutritional content increased by increasing the micronutrient rates under the same rate of amino acids. The highest value of total chlorophyll was recorded as 10.86 due to A2M2, while the lowest one was 7.98 due to A0M0, with an increase of 36% in the first season. For the total carbohydrates, the highest value was 13.17% due to the application of A2M2, while the lowest one was 10.99% due to A0M0, with an increase of 19.8% in the first season. The results of the C/N ratio reveal that increasing the amino acids rate in the presence of a micronutrient mixture did not affect the C/N ratio significantly. The highest nitrogen content was 17.27 which was attributed to A2M2, while the lowest one was 14 due to A0M0 with an increase of 23.4% in the first season; however, there was no significant interaction in the second season. For the plant content of phosphorus, increasing the phosphorus content was associated with increasing the amino acid rate in the absence and presence of the micronutrient mixture (M0 and M1), except for the highest rate of the micronutrient mixture (M2) which resulted in a significant decrease in the phosphorus content in both seasons; the lowest P-content was induced by A0M0 in both seasons. The most effective interaction was attributed to A0M2 in the first season; however, there was no significant differences among A2M1, A0M2, and A1M2 in the second season. The highest potassium content was recorded at 14.93 due to A2M2, while the lowest one was 11.13 due to A0M0 with an increase of 34.1% in the first season. The highest magnesium content (6.45%) was obtained due to the application of A2M2, while the lowest one was 5.90 due to A0M0 with an increase of 9.3% in the first season; however, there was no significant interaction in the second season. The highest iron content was 134.6 due to application of A2M2, while the lowest one was 116.7 in the control with an increase of 15.3% in the first season. Likewise, the results in the second season show that the highest iron content (135.4%) was due to A2M2, while the lowest one was 117.6 due to A0M0 with an increase of 15.1%. The highest zinc content was 47.89 due to the application of A2M2 in the first season. Likewise, the highest zinc content (48.36%) was due to A2M2 in the second season. The lowest content of zinc was attributed to the absence of amino acids and the application of the micronutrients (A0M0) in both seasons and there was no significant difference between A0 and A1 in the absence of the micronutrient mixture (M).

### 2.2. Yield and Quality

The obtained results illustrate that increasing the amino acid rate resulted in a progressive increase pattern in the mango yield and the quality parameters in both seasons. The most effective rate was 2 mL L^−1^ (A2) in both seasons (first and second, respectively) for all the aforementioned traits which recorded significant increases of 5.9% and 6% in panicle length, 6.7% and 6.9% in panicle diameter (Table S6), 4.5% and 4.5% in fruit set, 13.6% and 13.6% in fruit retention, 8.6% and 9.7% in the number of fruits/tree (Table 2), 2.2% and 2.7% in fruit weight, 4.1% and 3.7% in firmness, 8.4% and 3.2% in pulp weight (Table 3), 8.6% and 9% in seed weight (Table S7), 10.9% and 12.6% in yield/tree and 10.8% and 12.6% in total yield (Table 4), 1.8% and 1.6% in T.T.S., and 8.9% and 8.5% in the T.T.S./acid ratio (Table 5), 3.9% and 3.8% in the total sugars as well as 4.2% and 3.9% in ascorbic acid (Table 6). On the other hand, there was a significant decrease in the acidity associated with increased rates of amino acid application in both seasons (Table 5). Moreover, the amino acid applications did not affect the peel weight significantly (Table S7). Concerning the C/N ratio, there was no significant difference between A0 and A1, whereas increasing the amino acids rate to A2 resulted in a significant decrease in both seasons. Increasing the micronutrient rates resulted in a progressive increase pattern in panicle length, panicle diameter, fruit set, fruit retention, number of fruits/tree, fruit weight, firmness, pulp weight, seed weight, T.T.S., total sugars, ascorbic acid, T.T.S./acid ratio, yield/tree and the total yield. The most effective rate was 2 g L^−1^ (M2) in both seasons for all the aforementioned traits which recorded significant increases of 13.9% and 13.4% in panicle length, 13.7% and 13.9% in panicle diameter, 13.9% and 14.3% in fruit set, 36.7% and 36.9% in fruit retention, 19.7% and 20% in the number of fruits/tree, 6.6% and 7.3% in fruit weight, 10.3% and 11.9% in firmness, 5.5% and 5.2% in pulp weight, 23.9% and 23.5% in seed weight, 3.4% and 3.2% in T.T.S., 8.4% and 8.5% in total sugars, 10.6% and 10.2% in ascorbic acid, 21.7% and 22.4% in T.T.S./acid ratio, 27.5% and 28.7% in yield/tree and 27.6% and 28.7% in total yield. On the other hand, there was a significant decrease in the acidity associated with increasing the rates of micronutrient application in both seasons. Moreover, the micronutrient applications did not affect the peel weight significantly. Concerning the C/N ratio, raising the rate of the amino acids to M2 resulted in a significant decrease in both seasons compared with M0.

The overall view is that the nutritional content increased by increasing the micronutrient rates under the same rate of amino acids. The results of the fruit set show that the highest values were 14.76% and 14.65% under the application of A2M2 with increases of 20.1% and 20.3% in the first and second seasons, respectively. Moreover, the results of the fruit retention show that the most effective treatment was A2M2, which recorded 3.22 and 3.14 with increases of 36.4% and 52.4% in the first and second seasons, respectively. The results of the number of the following traits of fruits per tree, fruit weight, seed weight and peel weight illustrate that there was no significant interaction between the treatments in both seasons. Concerning the firmness, the best interaction was A2M2 with increases of 14.4% and 15% in the first and second seasons, respectively. The significant increase in pulp weight was also attributed to A2M2, with increases of 9.8% and 9.5% in comparison with A0M0 in the first and second seasons, respectively. During the first season; the highest T.T.S. (%) was 18.73 due to A2M2, while the results of the second season show that increasing the rates of amino acids in the presence of the micro-nutrient mixture led to no significant increase in the T.S.S. values. Furthermore, increasing the amino acids rates in the presence of M2 had no significant implication on the total sugars in both seasons. The lowest value of acidity was 0.50 due to A2M2; however, the highest value was recorded in the absence of amino acids and the micronutrient application A0M0 in the first and second seasons and there was significant interaction in the second one. The results of ascorbic acid show that the highest values (28.73 and 28.89 mg 100 mL^−1^) were due to A2M2 with increases of 17.3% and 16.4% in the first and second seasons, respectively. The greatest ratio of T.T.S./acid was 37.24 due to A2M2 in the first season, while the results of the second season were non-significant. The results of yield/tree (kg) and yield (Mg ha^−1^) illustrate that there was no significant interaction in the first season, while the significant increase in the second season was attributed to A2M2 with increases of 46.6% and 46.6% for yield/tree (kg) and yield (Mg ha^−1^), respectively.

## 3. Discussion

Mango (*Mangifera indica* L.) is one of the most common and oldest cultivated fruits in tropical and subtropical regions spanning over 100 countries, but improving the yield and quality remains a significant challenge, particularly in arid regions [25,26]. Several experimental studies have been conducted to improve the growth and yield of mangoes, including the use of plant growth regulators and micronutrients [27], plant bioregulators [28,29], sorbitol [30], as well as foliar and soil applications of zinc and boron [1]. Nonetheless, the integration of micronutrients and amino acids with varying concentrations has received less attention thus far, confirming the importance of the current study.

The application of amino acids is a prevalent practice for horticultural crops all over the world, with the bulk of treatments involving bio stimulants containing a combination of amino acids [31]. However, the effectiveness of AA in increasing yield and quality is primarily determined by the type of amino acids as well as the plant and cultivar type [31,32,33]. The enhancement of the vegetative growth due to the application of amino acids has been attributed to their ability to improve the efficiency of nitrogen uptake [20,34]. In addition, amino acids constitute an eminent bio-stimulant that affects physiological activities directly or indirectly that influence plant growth and thereby yield and productivity [20]. Furthermore, amino acids are required for the biosynthesis of a variety of non-proteinic nitrogenous substances such as coenzymes, pigments, purine, vitamins, and pyrimidine bases. Many studies have explored the effect of the application of foliar amino acids on yield and quality for different crops [35,36,37,38]. However, less attention has been paid to studying the effects of AA on horticulture crops, particularly mango, in arid regions. The foliar application of a micronutrient mixture also improved the yield and quality of mangoes due to its significant role in improving nitrogen assimilation and photosynthesis activity [39,40,41]. The integration between the amino acids and the micronutrient mixtures has received less attention so far, and thus was considered in our study. The increased fruit weight in this study could be attributed to zinc, which is required for starch formation, and iron, which improves cell division and enlargement, as well as boron, which is actively involved in carbohydrate transport in plants. Thus, the higher rate of the combined micronutrient mixture (Fe + Mn + Zn + Cu + B) resulted in a higher yield and better quality of the fruit. Another mechanism that affects the higher yield induced by the application of micronutrients is related to the mobility of photo assimilates to fruits, as well as the contribution of cell expansion and division, which results in higher fruit weight in treated plants [42]. Increased fruit retention, on the other hand, could be attributed to boron, which is important in pollen germination and pollen tube growth, which is associated with improved pollination, fertilization, and fruit setting [43]. The increased number of fruits per tree could be attributed to the use of Zn, Fe, and B. When micronutrients are sprayed alone or in combination, they directly participate in various physiological processes and enzymatic activity, resulting in a greater accumulation of food materials and, ultimately, an increased yield [44]. Boron’s involvement in the hormonal metabolism, which increases cell division and expansion, could explain the increase in fruit weight in response to its application [45]. Furthermore, zinc plays a direct role in growth, and boron promotes the rapid mobilization of water and sugar in the fruit. This demonstrates the significance of combining micronutrient elements and amino acids to increase the yield and quality of mangoes.

**Figure 1:**
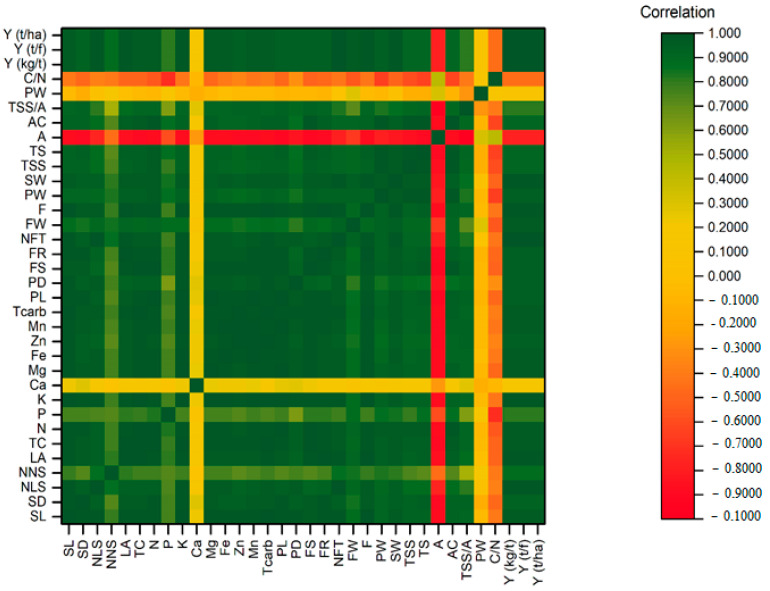
Correlation heatmap of the studied parameters of yield, nutritional status, and quality. The parameters are shoot length (SL), shoot diameter (SD), number of leaves per shoot (N/S), number of new shoots (NNS), leaf area (LA), total chlorophyll (TC), nitrogen (N), phosphorus (P), potassium (K), calcium (Ca), magnesium (Mg), iron (Fe), zinc (Zn), manganese (Mn), total carbohydrates (Tcarb), panicle length (PL), panicle diameter (PD), fruit set (FS), fruit retention (FR), No. fruits per tree (NFT), fruit weight (FW), firmness (F), pulp weight (PW), seed weight (SW), total soluble solid (TSS), total sugar (TS), acidity (A), ascorbic acid (AC), TSS/acid ratio, peel wight (Pw), C/N ratio, yield per tree (Y kg/t), yield per feddan (Y t/fed), and yield per hectare (Y t/ha).

**Figure 2:**
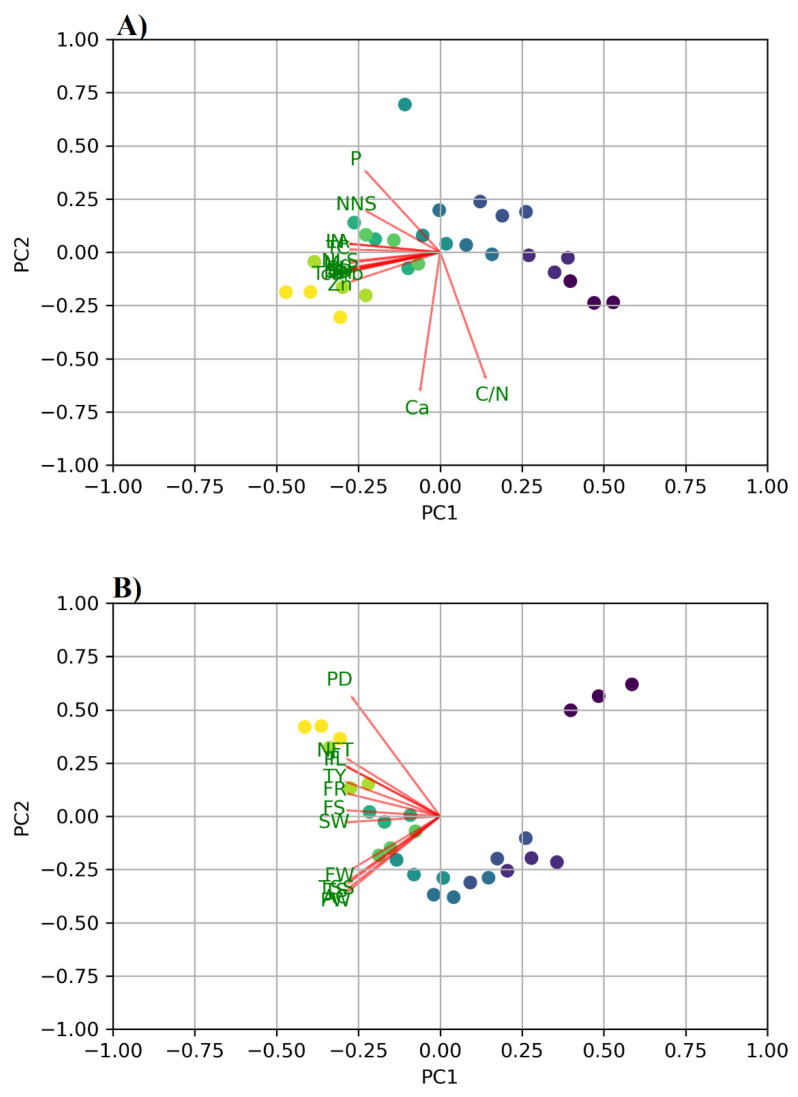
Principal Component Analysis (PCA) to show the correlation between treatments (scores) and crop parameters (vectors) for the vegetative growth and nutritional status (**A**), as well as the yield and quality (**B**). The score gradient colors from dark blue for A1M1 (without amino acids and micronutrients), light blue for A2M2 (1.0 mg L^−1^ amino acids, and 1.0 g L^−1^ micronutrients) and green to yellow for A3M3 (2.0 mg L^−1^ amino acids, and 2.0 g L^−1^ micronutrients). The loadings included shoot length (SL), shoot diameter (SD), number of leaves per shoot (N/S), number of new shoots (NNS), leaf area (LA), total chlorophyll (TC), nitrogen (N), phosphorus (P), potassium (K), calcium (Ca), magnesium (Mg), iron (Fe), zinc (Zn), manganese (Mn), total carbohydrates (Tcarb), panicle length (PL), panicle diameter (PD), fruit set (FS), fruit retention (FR), No. fruits per tree (NFT), fruit weight (FW), firmness (F), pulp weight (PW), seed weight (SW), total soluble solid (TSS), total sugar (TS), acidity (A), ascorbic acid (AC), TSS/acid ratio, peel wight (Pw), C/N ratio, yield per tree (Y kg/t), and yield per hectare (Y t/ha).

The heatmap correlation (Figure 1) revealed a positive correlation with all features except Ca, acidity, pulp weight, and the C/N ratio, allowing us to better understand the relationships between the yield, nutritional, and quality parameters. The PCA has also confirmed this relationship (Figure 2). This could be attributed to trees consuming more carbohydrates and calcium in order to increase the yield and quality. Furthermore, the Ca mineral is the major mineral contributor to the seed and pulp of mangoes, with a higher content in the seed than in the pulp [46]. The PCA summarized the relationships between yield, nutrition, and quality parameters, as well as the treatments. The PC1 contributed to a major component of the variations (84.8%), while PC2 contributed only 5.2% from the total variation. Except for acidity, C/N ratio, Ca, and pulp weight, the yield, nutrition status, and quality parameters had positive correlations with each other and with treatments A1M2, A1M3, A2M2, A2M3, A3M2, and A3M3. These treatments exhibited a negative correlation with A1M1, A2M1, and A3M1, while exhibiting a positive correlation with acidity, C/N ratio, and pulp weight (Figure 1). This demonstrates the significance of incorporating higher rates of micronutrient mixture and amino acids in increasing the yield, nutritional status, and quality of mangoes in arid regions.

## 4. Materials and Methods

### 4.1. Study Site, Design and Agronomic Practices

**Figure 3:**
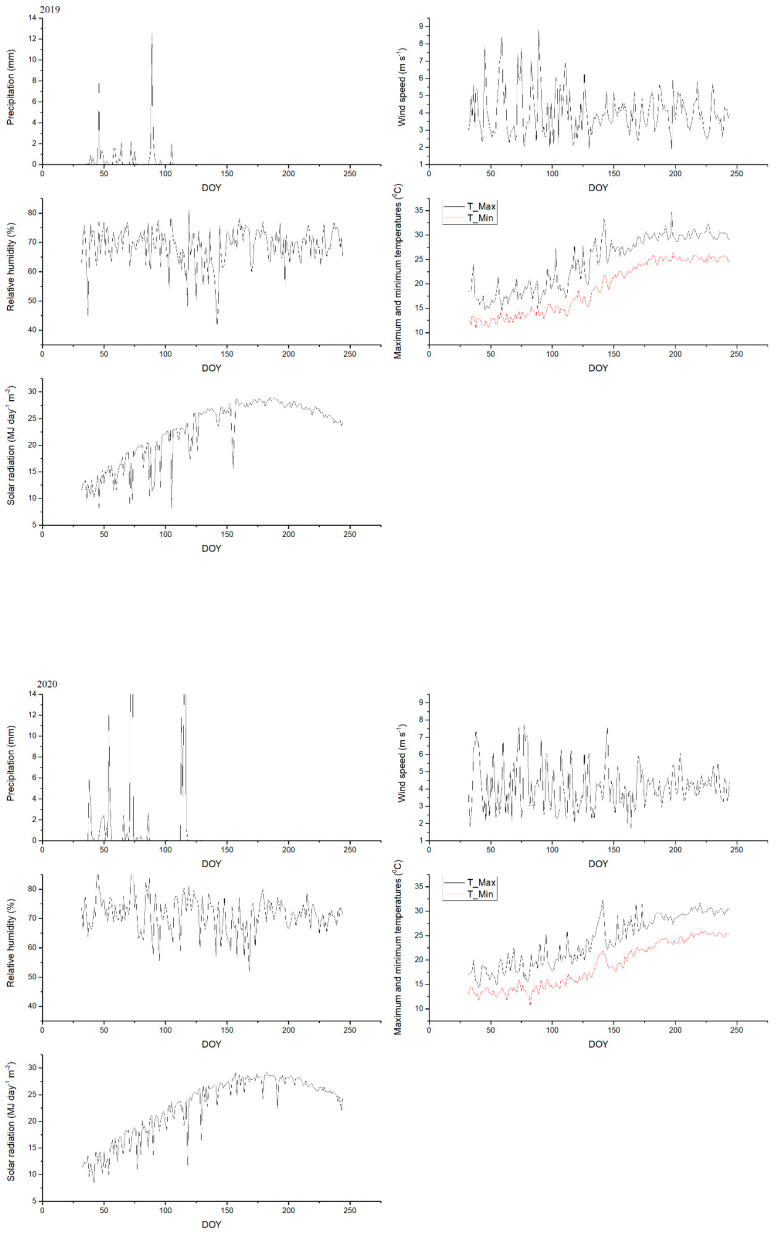
Daily climatic data as precipitation, wind speed, relative humidity, maximum and minimum temperatures, and solar radiation over two seasons (2019 and 2020).

This investigation was conducted through the 2019 and 2020 seasons on mango trees (cv. Fagri Kalan) in the El-Nobaria region, Egypt (31.2 N, and 29.9 E, and mean altitude 14 m above sea level). Twenty-seven Fagri Kalan mango trees that were 15 years old and were planted 55 m apart in sandy soil with a drip irrigation system were carefully selected to be as healthy, disease-free, and uniform in vigour and size as possible. The climatic data of the studied area over two growing seasons are presented in Figure 3. The purpose of this study was to investigate the effect of foliar spray with a micronutrient mixture (a commercial product containing 70.6, 42.0, 28.0, 20.0, and 6.0 g kg^−1^ for Zn, Mn, Fe, Cu, and B, respectively) and amino acids (Bioflow: commercial product containing 273 g L^−1^ amino acids) on the vegetative growth, leaf chemical constituents, productivity, and fruit quality of mango trees. The other horticultural practices such as pest management, irrigation, and pruning used in the region’s mango orchards were applied to all devoted trees on a regular basis according to the recommendations of the Ministry of Agriculture and Land Reclamation in Egypt. The experimental design was a two-factor factorial randomized complete block (RCBD) with three replicates for each treatment. The factors included three rates of the amino acids (AA) at the rates of 0.0, 1.0, and 2.0 mg L^−1^ represented as A_0_, A_1_ and A_2_, respectively, as well as three rates of the micronutrient mixture (M) at the rate of 0.0, 1.0, and 2.0 g L^−1^ (i.e M_0_, M_1_, and M_2_, respectively). As a result, the experiment included 9 treatments, each with 3 replicates, for a total of 27 trees under study. The soil texture was sandy, with a low fertility content, particularly in organic matter and microbial biomass (Table S8). All the investigated nutritional treatments, even in the control (water spray) were applied following the application of N, P, K fertilization according to the local recommendations. Chemical fertilizers such as N, P, and K were applied at rate of 210, 50 and 150 g per tree, respectively. During the 2019 and 2020 experimental seasons, chemical fertilizers were applied in two equal doses during the first week of February and two weeks later to the fruit, set via a drip irrigation system. Ammonium nitrate (33.5% N), phosphoric acid (60% P_2_O_5_), and potassium sulfate (50% K_2_O) were used as sources of N, P, and K fertilizers, respectively. Considering that spray treatments were applied to the entire foliage of each tree canopy, 5 L was found to be sufficient in this regard. All the applied treatments were sprayed four times during each growing season, namely at full bloom, fruit set, one and two months after fruit set, and before harvesting.

### 4.2. Measurements

#### 4.2.1. Vegetative Growth, Nutritional Status, and Yield

Four primary branches (limbs/scaffolds) of equal vigour that were well-distributed around the periphery of each tree (each facing one geographic direction) were carefully picked and identified in the third week of August. The number of new shoots, the shoot length, the shoot diameter, the number of leaves per shoot, and the leaf area were all determined using twelve spring cycle shoots. The total chlorophyll content in the fresh leaves was determined using a Minolta meter SPAD-502. During both seasons, representative samples of the fourth and fifth leaves from the base of the spring shoots were collected from each replicate in the second week of September. The samples were then washed with tap water, rinsed twice with distilled water, and oven-dried at 80 °C until they reached a consistent weight before being finely powdered for N, P, and K determinations. The total nitrogen in the leaf was determined by the modified micro Kjeldahl method mentioned by the authors of [47]. The total P in the leaf was determined after wet digestion using sulfuric and perchloric acid of plant leaves ground by the method of [48]. Meanwhile, the total K in the leaf was determined photometrically in the digested material according to the method described by the authors of [49]. Other elements such as Ca, Mg, Fe, Mn, and Zn, were determined using the Atomic absorption spectrophotometer “Perkin Elmer-3300” [50]. The total carbohydrates were determined photometrically in mature shoot dry samples (0.1 g) at 490 nm using the method described by [50,51]. The C/N ratio was estimated by dividing the total nitrogen in the leaf over the total carbohydrates in the shoot. To determine the initial number of fruits per panicle, the number of fruits per panicle was counted after about 15 days of full bloom. The fruit set was calculated as a percentage of perfect flowers using the equation below.

(1) $$\mathrm{Fruit}\;\mathrm{set}\;\left(\%\right)=\frac{\;\mathrm{The}\;\mathrm{average}\;\mathrm{number}\;\mathrm{of}\;\mathrm{fruitlets}\;\mathrm{per}\;\mathrm{panicle}}{\mathrm{The}\;\mathrm{average}\;\mathrm{number}\;\mathrm{of}\;\mathrm{perfect}\;\mathrm{flowers}\;\mathrm{per}\;\mathrm{panicle}}\;\times \;100$$

The fruit retention was counted for each of the tagged inflorescences at the beginning of the harvest according the following equation.

(2) $$\mathrm{Fruit}\;\mathrm{retention}\;\%\;=\frac{\mathrm{The}\;\mathrm{total}\;\mathrm{number}\;\mathrm{of}\;\mathrm{fruits}\;\mathrm{retained}\;}{\mathrm{Total}\;\mathrm{number}\;\mathrm{of}\;\mathrm{setting}\;\mathrm{fruits}}\;\times 100$$

The tree yield was estimated as the weight of the harvested fruits (kg tree^−1^ and t ha^−1^) in the 1st week of August in each season.

#### 4.2.2. Fruit Quality (Physical and Chemical Properties)

Twenty fruits were selected at random from each tree’s yield and transported to the laboratory to determine the physical and chemical properties of the fruits. The average fruit weight was determined by weighing a sample of twenty fruits from each replicate and calculating the average fruit weight (g). Fruit firmness was determined on three fruits per replicate, then the three measurements were taken from each fruit using a Push pull dynamometer (Model FT327) with pluger tip 5/16. The average firmness of the sample was expressed as Lb/inch^2^. The average seed weight was determined by weighing a sample of twenty seeds from each replicate and calculating the average seed, pulp, and peel weight (g). The percentage of the total soluble solids was measured refractometrically according to the authors of [46,52] using a hand refractometer ATAGO, Japan (Oto 32%). The TSS/acid ratio was estimated by dividing the total percentage of soluble solids over the total acidity percentage. Ascorbic acid was determined by titration against 2,6-di-chloro-phenol indo- phenol and calculated as (mg L^−1^ ascorbic acid/100 g pulp) [53] The total sugars in the fruit pulp were determined calorimetrically according to the method described by the authors of [52]

### 4.3. Statistical Analysis

All data parameters studied were analyzed as Factorial Completely Randomized Designs in factorial arrangement with three replications and subjected to a statistical analysis described by the authors of [53]. Significant differences among the means of various treatments were compared by the least significant difference (LSD) at a 5% level of significance using MSTAT-C. In addition, the Principal Component Analysis (PCA) for the yield and quality parameters under the corresponding treatments and the correlation heatmap were performed using the seaborn library in python.

## 5. Conclusions

The integration of a micronutrient mixture and amino acids with different rates significantly improved the yield, nutritional status, and quality of mangoes in arid regions with low fertility soils. The higher rates of the micronutrient mixture (2 g L^−1^) in combination with the highest amino acid concentration (2 mg L^−1^) were superior and could be recommended to improve the nutritional status, productivity, and quality of Fagri Kalan mango trees under similar environmental conditions and horticulture practices used in the current experiment. Nevertheless, using a broad rate of these treatments and different forms (i.e., chelated and nano) on different cultivars could enhance the yield and quality under biotic stress conditions.

## Figures and Tables

**Table 1:** The effect of the micronutrient mixture and the amino acid treatments and their combinations on the stem content of total chlorophyll, total carbohydrates, and the C/N ratio of mangoes during the 2019 and 2020 seasons.

Total Chlorophyll (mg/100 g Fresh Weight)								
Mix rate (g L^−1^)	1st Season				2nd Season			
	Amino acids rate (mg L^−1^)			Mean	Amino acids rate (mg L^−1^)			Mean
	A0 (without)	A1 (Conc.)	A2 (Conc.)		A0 (without)	A1 (Conc.)	A2 (Conc.)	
M0 (without)	7.98	8.29	8.85	8.37	8.36	8.70	9.20	8.75
M1 (Conc.)	9.28	9.89	10.07	9.75	9.79	10.28	10.54	10.20
M2 (Conc.)	10.01	10.33	10.86	10.40	10.85	10.98	11.50	11.11
Mean	9.09	9.50	9.93		9.67	9.98	10.41	
LSD (0.05)	M = 0.05 A = 0.05 M × A = 0.08				M = 0.13 A = 0.13 M × A = ns			
Total carbohydrates %/stem								
Micro nutrient mixture rate (g L^−1^)	1st Season				2nd Season			
	Amino acids rate (mg L^−1^)			Mean	Amino acids rate (mg L^−1^)			Mean
	A0 (without)	A1 (Conc.)	A2 (Conc.)		A0 (without)	A1 (Conc.)	A2 (Conc.)	
M0 (without)	10.99	11.27	11.41	11.22	11.30	11.63	11.73	11.55
M1 (Conc.)	11.93	12.18	12.45	12.19	12.25	12.49	12.81	12.52
M2 (Conc.)	12.36	12.85	13.17	12.79	12.81	13.19	13.52	13.17
Mean	11.76	12.10	12.35		12.12	12.43	12.69	
LSD (0.05)	M = 0.04 A = 0.04 M × A = 0.06				M = 0.06 A = 0.06 M × A = 0.10			
C/N ratio/stem								
Micro nutrient mixture rate (g L^−1^)	1st Season				2nd Season			
	Amino acids rate (mg L^−1^)			Mean	Amino acids rate (mg L^−1^)			Mean
	A0 (without)	A1 (Conc.)	A2 (Conc.)		A0 (without)	A1 (Conc.)	A2 (Conc.)	
M0 (without)	0.785	0.770	0.749	0.768	0.777	0.782	0.737	0.765
M1 (Conc.)	0.759	0.757	0.753	0.756	0.736	0.746	0.743	0.742
M2 (Conc.)	0.757	0.764	0.763	0.761	0.755	0.753	0.750	0.753
Mean	0.767	0.764	0.755		0.756	0.760	0.743	
LSD (0.05)	M = 0.003 A = 0.003 M × A = 0.006				M = 0.01 A = 0.01 M × A = 0.02			

**Table 2:** The effect of the micronutrient mixture, the amino acid treatments, and their combinations on fruit set % and fruit retention/panicle of mangoes during the 2019 and 2020 seasons.

Fruit Set (%)								
Mix rate (g L^−1^)	1st Season				2nd Season			
	Amino acids rate (mg L^−1^)			Mean	Amino acids rate (mg L^−1^)			Mean
	A0 (without)	A1 (Conc.)	A2 (Conc.)		A0 (without)	A1 (Conc.)	A2 (Conc.)	
M0 (without)	12.29	12.69	13.11	12.70	12.18	12.55	12.99	12.57
M1 (Conc.)	13.62	13.87	14.04	13.85	13.52	13.73	13.95	13.73
M2 (Conc.)	14.20	14.42	14.76	14.46	14.11	14.34	14.65	14.37
Mean	13.37	13.66	13.97		13.27	13.54	13.87	
LSD (0.05)	M = 0.03 A = 0.03 M × A = 0.06				M = 0.05 A = 0.05 M × A = 0.08			
Fruit retention/panicle								
Mix rate (g L^−1^)	1st Season				2nd Season			
	Amino acids rate (mg L^−1^)			Mean	Amino acids rate (mg L^−1^)			Mean
	A0 (without)	A1 (Conc.)	A2 (Conc.)		A0 (without)	A1 (Conc.)	A2 (Conc.)	
M0 (without)	2.14	2.30	2.44	2.29	2.06	2.22	2.37	2.22
M1 (Conc.)	2.60	2.94	3.10	2.88	2.54	2.88	3.02	2.81
M2 (Conc.)	2.98	3.18	3.22	3.13	2.89	3.10	3.14	3.04
Mean	2.57	2.81	2.92		2.50	2.73	2.84	
LSD (0.05)	M = 0.03 A = 0.03 M × A = 0.05				M = 0.03 A = 0.03 M × A = 0.06			
No. of fruits/tree								
Micro nutrient mixture rate (g L^−1^)	1st Season				2nd Season			
	Amino acids rate (mg L^−1^)			Mean	Amino acids rate (mg L^−1^)			Mean
	A0 (without)	A1 (Conc.)	A2 (Conc.)		A0 (without)	A1 (Conc.)	A2 (Conc.)	
M0 (without)	59.33	61.33	63.33	61.33	59.00	61.67	64.33	61.67
M1 (Conc.)	65.67	68.67	71.33	68.56	66.67	70.00	72.67	69.78
M2 (Conc.)	69.67	74.00	76.67	73.44	70.33	73.67	78.00	74.00
Mean	64.89	68.00	70.44		65.33	68.44	71.67	
LSD (0.05)	M = 0.53 A = 0.53 M × A = ns				M = 0.76 A = 0.76 M × A = ns			

M0 and A0: control, A1: amino acids with concentration of 0.1 mL L^−1^, A2: amino acids with rate of 2.0 mL L^−1^, M1: micronutrient mixture with 1.0%, M2: micronutrient mixture with 2.0%.

**Table 3:** The effect of the micronutrient mixture, the amino acid treatments, and their combinations on fruit weight, firmness, pulp weight, seed weight, and peel weight of mangoes during the 2019 and 2020 seasons.

Fruit Weight (g)								
Mix rate (g L^−1^)	1st Season				2nd Season			
	Amino acids rate (mg L^−1^)			Mean	Amino acids rate (mg L^−1^)			Mean
	A0 (without)	A1 (Conc.)	A2 (Conc.)		A0 (without)	A1 (Conc.)	A2 (Conc.)	
M0 (without)	513.7	528.3	534.7	525.6	501.7	514.7	526.0	514.1
M1 (Conc.)	548.0	552.7	554.0	551.6	536.7	543.7	546.3	542.2
M2 (Conc.)	555.7	560.7	564.3	560.2	547.7	550.7	556.7	551.7
Mean	539.1	547.2	551.0		528.7	536.3	543.0	
LSD (0.05)	M = 3.94 A = 3.94 M × A = ns				M = 4.32 A = 4.32 M × A = ns			
Firmness (Lb/inch^2^)								
Mix rate (g L^−1^)	1st Season				2nd Season			
	Amino acids rate (mg L^−1^)			Mean	Amino acids rate (mg L^−1^)			Mean
	A0 (without)	A1 (Conc.)	A2 (Conc.)		A0 (without)	A1 (Conc.)	A2 (Conc.)	
M0 (without)	2.29	2.34	2.37	2.34	2.32	2.37	2.40	2.36
M1 (Conc.)	2.41	2.50	2.53	2.48	2.45	2.54	2.58	2.52
M2 (Conc.)	2.52	2.59	2.62	2.58	2.60	2.63	2.67	2.64
Mean	2.41	2.48	2.51		2.46	2.51	2.55	
LSD (0.05)	M = 0.007 A = 0.007 M × A = 0.013				M = 0.01 A = 0.01 M × A = 0.02			
Pulp weight (g)								
Mix rate (g L^−1^)	1st Season				2nd Season			
	Amino acids rate (mg L^−1^)			Mean	Amino acids rate (mg L^−1^)			Mean
	A0 (without)	A1 (Conc.)	A2 (Conc.)		A0 (without)	A1 (Conc.)	A2 (Conc.)	
M0 (without)	424.3	440.3	447.3	437.3	421.0	437.0	443.7	433.9
M1 (Conc.)	452.7	458.0	461.0	457.2	447.7	452.0	456.7	452.1
M2 (Conc.)	456.0	462.3	466.0	461.4	451.0	457.3	461.0	456.4
Mean	444.3	453.6	458.1		439.9	448.8	453.8	
LSD (0.05)	M = 1.14 A = 1.14 M × A = 1.97				M = 1.56 A = 1.56 M × A = 2.71			

**Table 4:** The effect of the micronutrient mixture, the amino acid treatments, and their combinations on yield tree/kg and yield/(Mg/h^−1^) of mangoes during the 2019 and 2020 seasons.

Yield/Tree (kg)								
Micro nutrient mixture rate (g L^−1^)	1st Season				2nd Season			
	Amino acids rate (mg L^−1^)			Mean	Amino acids rate (mg L^−1^)			Mean
	A0 (without)	A1 (Conc.)	A2 (Conc.)		A0 (without)	A1 (Conc.)	A2 (Conc.)	
M0 (without)	30.50	32.41	33.89	32.27	29.62	31.74	33.86	31.74
M1 (Conc.)	36.01	37.97	39.54	37.84	35.80	38.07	39.71	37.86
M2 (Conc.)	38.72	41.50	43.27	41.16	38.53	40.58	43.43	40.85
Mean	35.08	37.29	38.90		34.65	36.80	39.00	
LSD (0.05)	M = 0.40 A = 0.40 M × A = ns				M = 0.45 A = 0.45 M × A = 0.84			
Yield (Mg ha^−1^)								
Micro nutrient mixture rate (g L^−1^)	1st Season				2nd Season			
	Amino acids rate (mg L^−1^)			Mean	Amino acids rate (mg L^−1^)			Mean
	A0 (without)	A1 (Conc.)	A2 (Conc.)		A0 (without)	A1 (Conc.)	A2 (Conc.)	
M0 (without)	12.20	12.96	13.55	12.90	11.84	12.69	13.54	12.69
M1 (Conc.)	14.40	15.18	15.81	15.13	14.32	15.22	15.88	15.14
M2 (Conc.)	15.48	16.59	17.30	16.46	15.40	16.23	17.36	16.33
Mean	14.03	14.91	15.55		13.85	14.71	15.59	
LSD (0.05)	M = 0.16 A = 0.16 M × A = ns				M = 0.20 A = 0.20 M × A = 0.34			

**Table 5:** The effect of the micronutrient mixture, the amino acid treatments, and their combinations on the TSS, acidity and T.T.S./acid ratio of mango cultivar during the 2019 and 2020 seasons.

T.T.S. (%)								
Mix rate (g L^−1^)	1st Season				2nd Season			
	Amino acids rate (mg L^−1^)			Mean	Amino acids rate (mg L^−1^)			Mean
	A0 (without)	A1 (Conc.)	A2 (Conc.)		A0 (without)	A1 (Conc.)	A2 (Conc.)	
M0 (without)	17.64	18.15	18.18	17.99	17.92	18.32	18.49	18.24
M1 (Conc.)	18.32	18.41	18.48	18.40	18.56	18.73	18.74	18.68
M2 (Conc.)	18.48	18.61	18.73	18.61	18.78	18.83	18.88	18.83
Mean	18.15	18.39	18.47		18.42	18.63	18.71	
LSD (0.05)	M = 0.03 A = 0.03 M × A = 0.05				M = 0.06 A = 0.06 M × A = 0.11			
Acidity (%)								
Mix rate (g L^−1^)	1st Season				2nd Season			
	Amino acids rate (mg L^−1^)			Mean	Amino acids rate (mg L^−1^)			Mean
	A0 (without)	A1 (Conc.)	A2 (Conc.)		A0 (without)	A1 (Conc.)	A2 (Conc.)	
M0 (without)	0.64	0.61	0.59	0.61	0.63	0.59	0.57	0.59
M1 (Conc.)	0.59	0.56	0.54	0.56	0.56	0.54	0.52	0.54
M2 (Conc.)	0.54	0.52	0.50	0.52	0.51	0.50	0.49	0.50
Mean	0.59	0.56	0.55		0.57	0.54	0.53	
LSD (0.05)	M = 0.003 A = 0.003 M × A = 0.005				M = 0.009 A = 0.009 M × A = ns			
T.T.S./acid ratio								
Mix rate (g L^−1^)	1st Season				2nd Season			
	Amino acids rate (mg L^−1^)			Mean	Amino acids rate (mg L^−1^)			Mean
	A0 (without)	A1 (Conc.)	A2 (Conc.)		A0 (without)	A1 (Conc.)	A2 (Conc.)	
M0 (without)	27.43	29.93	30.82	29.39	28.60	31.23	32.45	30.76
M1 (Conc.)	31.08	33.08	34.03	32.73	33.06	34.95	35.89	34.63
M2 (Conc.)	34.46	35.58	37.24	35.76	36.89	37.44	38.57	37.64
Mean	30.99	32.87	34.03		32.85	34.54	35.64	
LSD (0.05)	M = 0.23 A = 0.23 M × A = 0.39				M = 0.68 A = 0.68 M × A = ns			

**Table 6:** The effect of the micronutrient mixture, the amino acid treatments, and their combinations on the total sugars and ascorbic acid of mangoes during the 2019 and 2020 seasons.

Total Sugar (%)								
Mix rate (g L^−1^)	1st Season				2nd Season			
	Amino acids rate (mg L^−1^)			Mean	Amino acids rate (mg L^−1^)			Mean
	A0 (without)	A1 (Conc.)	A2 (Conc.)		A0 (without)	A1 (Conc.)	A2 (Conc.)	
M0 (without)	12.06	12.80	13.00	12.63	12.26	12.95	13.21	12.81
M1 (Conc.)	13.10	13.39	13.51	13.33	13.34	13.59	13.71	13.55
M2 (Conc.)	13.59	13.73	13.75	13.69	13.82	13.89	13.99	13.90
Mean	12.92	13.31	13.42		13.14	13.48	13.64	
LSD (0.05)	M = 0.03 A = 0.03 M × A = 0.06				M = 0.07 A = 0.07 M × A = 0.12			
Ascorbic acid (mg 100 mL^−1^)								
Mix rate (g L^−1^)	1st Season				2nd Season			
	Amino acids rate (mg L^−1^)			Mean	Amino acids rate (mg L^−1^)			Mean
	A0 (without)	A1 (Conc.)	A2 (Conc.)		A0 (without)	A1 (Conc.)	A2 (Conc.)	
M0 (without)	24.50	26.05	26.60	25.72	24.83	26.19	26.88	25.97
M1 (Conc.)	27.56	27.88	28.14	27.86	27.83	28.02	28.33	28.06
M2 (Conc.)	28.07	28.54	28.73	28.45	28.26	28.67	28.89	28.61
Mean	26.71	27.49	27.82		26.97	27.63	28.03	
LSD (0.05)	M = 0.07 A = 0.07 M × A = 0.13				M = 0.09 A = 0.09 M × A = 0.15			

## Data Availability

The data supporting reported results can be found here and in the Supplementary Materials.

## Supplementary Materials

The following are available online at https://www.mdpi.com/article/10.3390/plants10102057/s1, Table S1: The effect of the micronutrient mixture and the amino acid treatments and their combinations on shoot length, shoot diameter and the number of new shoots, the number of leaves/shoot and leaf area of mango during the 2019 and 2020 seasons, Table S2: The effect of the micronutrient mixture and the amino acid treatments and their combinations on the number of leaves/shoot and leaf area (cm^2^) of “Fagary Kalan” mango cultivar during the 2019 and 2020 seasons, Table S3: The effect of the micronutrient mixture and the amino acid treatments and their combinations on leaf nitrogen, phosphorus and potassium contents of “Fagary Kalan” mango cultivar during the 2019 and 2020 seasons, Table S4: The effect of the micronutrient mixture and the amino acid treatments and their combinations on the leaf calcium and magnesium contents of “Fagary Kalan” mango cultivar during the 2019 and 2020 seasons, Table S5: The effect of the micronutrient mixture and the amino acid treatments and their combinations on leaf micro nutrients of “Fagary Kalan” mango cultivar during the 2019 and 2020 seasons, Table S6: The effect of the micronutrient mixture, the amino acid treatments, and their combinations on panicle length and diameter (cm) of “Fagary Kalan” mango cultivar during the 2019 and 2020 seasons, Table S7: The effect of the micronutrient mixture, the amino acid treatments, and their combinations on seed weight (g) and peel weight (g) of “Fagary Kalan” mango cultivar during the 2019 and 2020 seasons, Table S8: Initial soil physiochemical properties before the cultivation in the first growing season.
