# Supplementary material for: The Exogenous Application of Micro-Nutrient Elements and Amino Acids Improved the Yield, Nutritional Status and Quality of Mango in Arid Regions
Source: Plants (Basel). 2021 Sep 29;10(10):2057. doi: 10.3390/plants10102057 (PMC8540748; doi:10.3390/plants10102057)
Supplement: Supplementary file 1 [file plants-10-02057-s001.zip › plants-1373648-supplementary.pdf]

Supplementary

The Exogenous Application of Micro-Nutrient Elements and Amino Acids Improved the Yield, Nutritional Status and Quality of Mango in Arid Regions

Ahmed M. S. Kheir [1,2], Zheli Ding [1,*], Mohamed S. Gawish [3], Hanan M. Abou El Ghit [4], Taghred A. Hashim [5], Esmat F. Ali [6], Mamdouh A. Eissa [7], Zhaoxi Zhou [1,*], Mohammad S. Al-Harbi [6] and Sherif Fathy El-Gioushy [8,*]

1 Haikou Experimental Station, Chinese Academy of Tropical Agricultural Sciences (CATAS), Haikou 570000, China; ahmedkheir@arc.sci.eg
2 Agricultural Research Center, Soils, Water and Environment Research Institute, Giza, 12112, Egypt
3 Pomology Department, Faculty of Agriculture, Damietta University, Damietta Governorate, 34511, Egypt; msagawishaa@gmail.com
4 Botany and Microbiology Department, Faculty of Science, Helwan University, Cairo Governorate 11111, Egypt; hanan8760@yahoo.com
5 Soil and Water Department, Faculty of Agriculture (Moshtohor), Benha University, Moshtohor, Toukh, 13736, Egypt; taghreed.hashem@fagr.bu.edu.eg
6 Biology Department, Faculty of Science, Taif University, Taif 26571, Saudi Arabia; a.esmat@tu.edu.sa (E.F.A.); mharbi@tu.edu.sa (M.S.A-.H.)
7 Department of Soils and Water, Faculty of Agriculture, Assiut University, Assiut 71526, Egypt; mamdouh.eisa@aun.edu.eg
8 Horticulture Department, Faculty of Agriculture (Moshtohor), Benha University, Moshtohor, Toukh, 13736, Egypt
* Correspondence: dingzheli@zju.edu.cn (Z.D.); zhouzhaoxi@catas.cn (Z.Z.); sherif.elgioushy@fagr.bu.edu.eg (S.F.E.)

Table S1. Effect of micronutrients mixture and amino acids treatments and its combinations on shoot length, shoot diameter and number of new shoots, number of leaves/ shoot and leaf area of mango during 2019 and 2020 seasons.

			**Shoot Length (cm)**					
	1st Season				2nd Season			
Mix rate	Amino acid rate (mg L^{-1})				Amino acid rate (mg L^{-1})			
(g L^{-1})	A_0 (without)	A_1 (Conc.)	A_2 (Conc.)	Mean	A_0 (without)	A_1 (Conc.)	A_2 (Conc.)	Mean
M_0 (without)	44.03	46.03	47.09	45.72	44.86	46.44	48.04	46.45
M_1 (Conc.)	48.36	51.49	54.37	51.40	48.97	51.78	54.97	51.90
M_2 (Conc.)	52.68	56.18	57.40	55.42	53.14	56.88	58.41	56.14
Mean	48.36	51.23	52.95		48.99	51.70	53.81	
LSD $_{(0.05)}$	M = 0.22	A = 0.22	M × A = 0.38		M = 0.34	A = 0.34	M × A = 0.59	
			**Shoot Diameter (cm)**					
	1st Season				2nd Season			
Mix rate	Amino acid rate (mg L^{-1})				Amino acid rate (mg L^{-1})			
(g L^{-1})	A_0 (without)	A_1 (Conc.)	A_2 (Conc.)	Mean	A_0 (without)	A_1 (Conc.)	A_2 (Conc.)	Mean
M_0 (without)	10.28	11.35	11.89	11.17	10.44	11.51	12.01	11.32
M_1 (Conc.)	12.30	12.72	14.73	13.25	12.42	12.85	14.82	13.36
M_2 (Conc.)	13.73	14.86	15.13	14.57	13.82	14.97	15.26	14.68
Mean	12.10	12.97	13.92		12.23	13.11	14.03	
LSD $_{(0.05)}$	M = 0.04	A = 0.04	M × A = 0.07		M = 0.12	A = 0.12	M × A = 0.21	
			**No. of New Shoot**					
	1st Season				2nd Season			
Mix rate	Amino acid rate (mg L^{-1})				Amino acid rate (mg L^{-1})			
(g L^{-1})	A_0 (without)	A_1 (Conc.)	A_2 (Conc.)	Mean	A_0 (without)	A_1 (Conc.)	A_2 (Conc.)	Mean
M_0 (without)	9.3	10.3	10.7	10.11	10.33	11.33	12.00	11.22
M_1 (Conc.)	11.3	12.7	13.0	12.33	12.33	13.33	14.33	13.33
M_2 (Conc.)	11.3	13.3	14.0	12.89	12.33	14.33	15.00	13.89
Mean	10.67	12.11	12.56		11.67	13.00	13.78	
LSD $_{(0.05)}$	M = 0.44	A = 0.44	M × A = ns		M = 0.60	A = 0.60	M × A = 1.03	

Table S2. Effect of micronutrients mixture and amino acids treatments and its combinations on number of leaves/shoot and leaf area (cm2) of "Fagary Kalan" mango cultivar during 2019 and 2020 seasons.

Mix rate (g L^{-1})	1st Season Amino acid rate (mg L^{-1})				2nd Season Amino acid rate (mg L^{-1})			
**No. of Leaves/Shoot**								
	A_0 (without)	A_1 (Conc.)	A_2 (Conc.)	Mean	A_0 (without)	A_1 (Conc.)	A_2 (Conc.)	Mean
M_0 (without)	33.00	34.33	35.67	34.33	34.33	36.00	37.33	35.89
M_1 (Conc.)	37.33	39.00	41.67	39.33	38.00	41.00	43.00	40.67
M_2 (Conc.)	39.67	45.00	46.00	43.56	40.67	46.33	48.33	45.11
Mean	36.67	39.44	41.11		37.67	41.11	42.89	
LSD $_{(0.05)}$	M = 0.46 A = 0.46 M × A = 0.8				M = 0.69 A = 0.69 M × A = 1.19			
**Leaf Area (cm^2)**								
Mix rate (g L^{-1})	1st Season Amino acid rate (mg L^{-1})				2nd Season Amino acid rate (mg L^{-1})			
	A_0 (without)	A_1 (Conc.)	A_2 (Conc.)	Mean	A_0 (without)	A_1 (Conc.)	A_2 (Conc.)	Mean
M_0 (without)	70.88	71.67	72.11	71.56	71.50	72.00	72.51	72.00
M_1 (Conc.)	73.18	74.17	74.71	73.97	73.38	74.63	75.40	74.47
M_2 (Conc.)	74.14	75.00	75.56	74.90	74.75	75.93	76.62	75.77
Mean	72.68	73.61	74.13		73.21	74.19	74.84	
LSD $_{(0.05)}$	M = 0.15 A = 0.15 M × A = 0.26				M = 0.20 A = 0.20 M × A = 0.34			

Table S3. Effect of micronutrients mixture and amino acids treatments and its combinations on leaf nitrogen, phosphorus and potassium contents of "Fagary Kalan" mango cultivar during 2019 and 2020 seasons.

Mix rate (g L^{-1})	1st Season Amino acid rate (mg L^{-1}) A_0 (without)	A_1 (Conc.)	A_2 (Conc.)	Mean	2nd Season Amino acid rate (mg L^{-1}) A_0 (without)	A_1 (Conc.)	A_2 (Conc.)	Mean
**Nitrogen Content (g kg^{-1})**								
M_0 (without)	14.00	14.63	15.23	14.62	14.55	14.88	15.92	15.12
M_1 (Conc.)	15.73	16.10	16.53	16.12	16.64	16.74	17.26	16.88
M_2 (Conc.)	16.33	16.83	17.27	16.81	16.97	17.51	18.03	17.50
Mean	15.37	15.86	16.34		16.05	16.37	17.07	
LSD $_{(0.05)}$	M = 0.05	A = 0.05	M × A = 0.09		M = 0.21	A = 0.21	M × A = ns	
**Phosphorus Content (g kg^{-1})**								
M_0 (without)	3.50	3.70	4.03	3.74	3.70	3.85	4.23	3.93
M_1 (Conc.)	4.13	4.20	4.30	4.21	4.34	4.48	4.65	4.49
M_2 (Conc.)	4.43	4.33	4.10	4.29	4.75	4.70	4.33	4.49
Mean	4.02	4.08	4.14		4.26	4.34	4.40	
LSD $_{(0.05)}$	M = 0.05	A = 0.05	M × A = 0.09		M = 0.08	A = 0.08	M × A = 0.14	
**Potassium Content (g kg^{-1})**								
M_0 (without)	11.13	11.53	12.13	11.60	11.42	11.92	12.47	11.94
M_1 (Conc.)	12.53	13.13	13.67	13.11	12.84	13.34	13.96	13.38
M_2 (Conc.)	13.53	14.43	14.93	14.30	12.88	14.79	15.27	14.65
Mean	12.40	13.03	13.58		12.71	13.35	13.90	
LSD $_{(0.05)}$	M = 0.05	A = 0.05	M × A = 0.08		M = 0.09	A = 0.09	M × A = 0.15	

Table S4. Effect of micronutrients mixture and amino acids treatments and its combinations on leaf calcium and magnesium contents of "Fagary Kalan" mango cultivar during 2019 and 2020 seasons.

**Calcium Content (g kg⁻¹)**								
	1st Season				2nd Season			
Mix rate (g L⁻¹)	Amino acid rate (mg L⁻¹)				Amino acid rate (mg L⁻¹)			
	A_0 (without)	A_1 (Conc.)	A_2 (Conc.)	Mean	A_0 (without)	A_1 (Conc.)	A_2 (Conc.)	Mean
M_0 (without)	19.30	19.97	20.47	19.91	19.67	20.40	20.87	20.31
M_1 (Conc.)	20.67	14.90	21.93	19.17	21.00	15.27	22.40	19.56
M_2 (Conc.)	21.30	22.47	22.77	22.18	21.67	22.83	23.17	22.56
Mean	20.42	19.11	21.72		20.78	19.50	22.14	
LSD $_{(0.05)}$	M = ns	A = ns	M × A = ns		M = ns	A = ns	M × A = ns	
**Magnesium content (g kg⁻¹)**								
	1st Season				2nd Season			
Mix rate (g L⁻¹)	Amino acid rate (mg L⁻¹)				Amino acid rate (mg L⁻¹)			
	A_0 (without)	A_1 (Conc.)	A_2 (Conc.)	Mean	A_0 (without)	A_1 (Conc.)	A_2 (Conc.)	Mean
M_0 (without)	5.90	5.94	6.10	5.98	6.22	5.90	6.48	6.20
M_1 (Conc.)	6.15	6.21	6.26	6.21	6.59	6.72	6.83	6.71
M_2 (Conc.)	6.24	6.40	6.45	6.37	6.85	6.94	6.98	6.92
Mean	6.10	6.18	6.27		6.55	6.52	6.76	
LSD $_{(0.05)}$	M = 0.008	A = 0.008	M × A = 0.014		M = 0.15	A = 0.15	M × A = ns	

Table S5. Effect of micronutrients mixture and amino acids treatments and its combinations on leaf micro nutrients of "Fagary Kalan" mango cultivar during 2019 and 2020 seasons.

**Iron Content (mg kg⁻¹)**								
	1st Season				2nd Season			
Mix rate	Amino acid rate (mg L⁻¹)				Amino acid rate (mg L⁻¹)			
(g L⁻¹)	A_0 (without)	A_1 (Conc.)	A_2 (Conc.)	Mean	A_0 (without)	A_1 (Conc.)	A_2 (Conc.)	Mean
M_0 (without)	116.7	118.7	121.0	118.8	117.6	119.2	121.8	119.5
M_1 (Conc.)	123.4	125.8	127.4	125.5	124.1	126.6	128.2	126.3
M_2 (Conc.)	130.2	132.6	134.6	132.5	130.9	133.1	135.4	133.1
Mean	123.5	125.7	127.7		124.2	126.3	128.5	
LSD $_{(0.05)}$	M = 0.43	A = 0.43	M × A = 0.75		M = 0.45	A = 0.45	M × A = 0.79	
**Zinc Content (mg kg⁻¹)**								
	1st Season				2nd Season			
Mix rate	Amino acid rate (mg L⁻¹)				Amino acid rate (mg L⁻¹)			
(g L⁻¹)	A_0 (without)	A_1 (Conc.)	A_2 (Conc.)	Mean	A_0 (without)	A_1 (Conc.)	A_2 (Conc.)	Mean
M_0 (without)	39.81	40.03	40.64	40.16	40.29	40.41	41.04	40.58
M_1 (Conc.)	42.83	43.95	44.85	43.88	43.18	44.39	45.25	44.27
M_2 (Conc.)	45.55	46.37	47.89	46.60	46.07	46.90	48.36	47.11
Mean	42.73	43.45	44.46		43.18	43.90	44.88	
LSD $_{(0.05)}$	M = 0.16	A = 0.16	M × A = 0.29		M = 0.17	A = 0.17	M × A = 0.29	
**Manganese Content (mg kg⁻¹)**								
	1st Season				2nd Season			
Mix rate	Amino acid rate (mg L⁻¹)				Amino acid rate (mg L⁻¹)			
(g L⁻¹)	A_0 (without)	A_1 (Conc.)	A_2 (Conc.)	Mean	A_0 (without)	A_1 (Conc.)	A_2 (Conc.)	Mean
M_0 (without)	44.64	45.35	46.27	45.42	45.30	45.70	46.67	45.89
M_1 (Conc.)	47.14	48.46	49.81	48.47	47.59	48.82	50.14	48.85
M_2 (Conc.)	50.36	50.98	52.01	51.12	50.71	51.35	52.34	51.46
Mean	47.38	48.27	49.37		47.87	48.62	49.72	
LSD $_{(0.05)}$	M = 0.20	A = 0.20	M × A = 0.35		M = 0.24	A = 0.24	M × A = 0.42	

Table S6. Effect of micronutrients mixture, amino acids treatments and its combinations on Panicle length and diameter (cm) of "Fagary Kalan" mango cultivar during 2019 and 2020 seasons.

**Panicle Length (cm)**								
	1st Season				2nd Season			
Mix rate	Amino acid rate (mg L^{-1})				Amino acid rate (mg L^{-1})			
(g L^{-1})	A_0 (without)	A_1 (Conc.)	A_2 (Conc.)	Mean	A_0 (without)	A_1 (Conc.)	A_2 (Conc.)	Mean
M_0 (without)	14.98	15.33	16.01	15.44	14.90	15.28	15.95	15.38
M_1 (Conc.)	16.21	16.53	17.16	16.63	16.14	16.45	17.08	16.56
M_2 (Conc.)	17.09	17.65	17.98	17.58	17.01	17.56	17.88	17.48
Mean	16.10	16.50	17.05		16.01	16.43	16.97	
LSD $_{(0.05)}$	M = 0.05	A = 0.05	M × A = 0.09		M = 0.07	A = 0.07	M × A = 0.12	
**Panicle Diameter (cm)**								
	1st Season				2nd Season			
Mix rate	Amino acid rate (mg L^{-1})				Amino acid rate (mg L^{-1})			
(g L^{-1})	A_0 (without)	A_1 (Conc.)	A_2 (Conc.)	Mean	A_0 (without)	A_1 (Conc.)	A_2 (Conc.)	Mean
M_0 (without)	9.07	9.19	9.39	9.22	8.94	9.08	9.29	9.10
M_1 (Conc.)	9.54	9.72	10.11	9.79	9.45	9.63	10.01	9.70
M_2 (Conc.)	9.93	10.56	10.96	10.48	9.81	10.46	10.84	10.37
Mean	9.51	9.82	10.15		9.40	9.72	10.05	
LSD $_{(0.05)}$	M = 0.05	A = 0.05	M × A = 0.09		M = 0.05	A = 0.05	M × A = 0.09	

Table S7. Effect of micronutrients mixture, amino acids treatments and its combinations on seed weight (g) and peel weight (g) of "Fagary Kalan" mango cultivar during 2019 and 2020 seasons.

Seed Weight (g)								
	1st Season				2nd Season			
Mix rate	Amino acid rate (mg L^{-1})				Amino acid rate (mg L^{-1})			
(g L^{-1})	A_0	A_1	A_2	Mean	A_0	A_1	A_2	Mean
	(without)	(Conc.)	(Conc.)		(without)	(Conc.)	(Conc.)	
M_0 (without)	41.0	45.3	46.3	44.22	39.00	43.87	44.28	42.38
M_1 (Conc.)	48.3	51.0	52.3	50.56	46.29	49.58	50.28	48.72
M_2 (Conc.)	53.3	55.0	56.0	54.78	50.83	52.45	53.78	52.35
Mean	47.56	50.44	51.56		45.37	48.63	49.45	
LSD $_{(0.05)}$	M = 0.66	A = 0.66	M × A = ns		M = 0.89	A = 0.89	M × A = ns	
**Peel Weight (g)**								
	1st Season				2nd Season			
Mix rate	Amino acid rate (mg L^{-1})				Amino acid rate (mg L^{-1})			
(g L^{-1})	A_0	A_1	A_2	Mean	A_0	A_1	A_2	Mean
	(without)	(Conc.)	(Conc.)		(without)	(Conc.)	(Conc.)	
M_0 (without)	48.33	42.67	41.00	44.00	41.67	33.80	38.05	37.84
M_1 (Conc.)	47.00	43.67	40.67	43.78	42.71	42.08	39.39	41.39
M_2 (Conc.)	46.33	43.33	42.33	44.00	45.84	40.88	41.88	42.87
Mean	47.22	43.22	41.33		43.41	38.92	39.77	
LSD $_{(0.05)}$	M = ns	A = ns	M × A = ns		M = ns	A = ns	M × A = ns	

Table S8. Initial soil physiochemical properties before the cultivation of first growing season.

**Soil Depth (cm)**	**EC (dS m^{-1})**	**pH**	**OM (%)**	**FC (%)**	**PWP (%)**
0–20	1.55	7.7	0.33	16.5	7.2
20–40	1.77	7.8	0.31	17.0	6.5
40–60	1.82	7.6	0.28	15.8	7.1
	Sand (%)	Silt (%)	Clay (%)	Soil texture	Bd (Mg m^{-3})
0–20	92.1	3.4	4.5	Sandy	1.44
20–40	91.5	2.7	5.8	Sandy	1.48
40–60	93.0	2.8	4.2	Sandy	1.52

EC: electrical conductivity, OM: organic matter content, FC: soil field capacity, PWP: permanent wilting point, Bd: soil bulk density.
